# The Safety and Efficacy of Inspiratory Muscle Training for Patients With Acute Myocardial Infarction Undergoing Percutaneous Coronary Intervention: Study Protocol for a Randomized Controlled Trial

**DOI:** 10.3389/fcvm.2020.598054

**Published:** 2021-01-12

**Authors:** YuanHui Liu, YiNing Dai, Zhi Liu, HuiMin Zhan, Manyu Zhu, XianYuan Chen, ShengQing Zhang, GuoLin Zhang, Ling Xue, ChongYang Duan, JiYan Chen, Lan Guo, PengCheng He, Ning Tan

**Affiliations:** ^1^Department of Cardiology, Guangdong Cardiovascular Institute, Guangdong Provincial Key Laboratory of Coronary Heart Disease Prevention, Guangdong Provincial People's Hospital, Guangdong Academy of Medical Sciences, Guangzhou, China; ^2^Department of Biostatistics, School of Public Health, Southern Medical University, Guangzhou, China

**Keywords:** pneumonia, inspiratory muscle training, acute myocardial infarction, percutaneous coronary intervention, intervention

## Abstract

**Background:** Uncommonly high rates of pneumonia in patients with acute myocardial infarction (AMI) undergoing primary percutaneous coronary intervention (PCI) have been observed during recent years. Inspiratory muscle training (IMT) could reduce pneumonia in patients undergoing coronary artery bypass grafting and other cardiac surgeries. The relationship between IMT and AMI is unknown. Here, we describe the feasibility and potential benefit of IMT in patients at high risk for pneumonia with AMI who have undergone primary PCI.

**Methods:** Our study is a prospective, randomized, controlled, single-center clinical trial. A total of 60 participants will be randomized into an IMT group and control group with 30 participants in each group. Participants in the IMT group will undergo training for 15 min *per session*, twice a day, from 12 to 24 h after primary PCI, until 30 days post-randomization; usual care will be provided for the control group. The primary endpoint is the change in inspiratory muscle strength, the secondary endpoint included feasibility, pneumonia, major adverse cardiovascular events, length of stay, pulmonary function tests measure, and quality of life.

**Discussion:** Our study is designed to evaluate the feasibility of IMT and its effectiveness in improving inspiratory muscle strength in participants with AMI who have undergone primary PCI.

**Clinical Trial Registration:** www.ClinicalTrials.gov, identifier: NCT04491760.

## Introduction

Uncommonly high rates (2–7%) of pneumonia have been reported in patients with acute myocardial infarction (AMI), thus increasing the mortality of such patients (1–4). However, other than antibiotic therapy, there are only a few treatment options against infection risk. Therefore, there is an urgent need for developing novel preventive strategies to decrease the risk of pneumonia in these patients. Maximal inspiratory pressure (MIP) is a simple, non-invasive way to evaluate respiratory muscle strength, and it is widely used clinically (5). Inspiratory muscle training (IMT) could significantly increase MIP and has been considered a feasible method to reduce pneumonia in patients undergoing cardiac surgery including coronary artery bypass grafting (6, 7). However, the effect of IMT on patients with AMI is poorly understood. Matos-Garcia and colleagues showed that respiratory muscle strength was impaired in patients with AMI compared to healthy subjects (8), and that such impairment might also later lead to pneumonia. To the best of our knowledge, no research has been performed to evaluate the value of IMT on patients with AMI. Therefore, we aim to conduct 30 days of IMT in patients with AMI who are at high risk of pneumonia and have undergone primary percutaneous coronary intervention (PCI), to evaluate the effectiveness and feasibility of IMT in this population.

## Materials and Methods

### Study Design

This proposed protocol involves a single-center, prospective, randomized study. The study is expected for enrollment from December 1st 2020 to April 30th 2021. All of the participants would be followed up until 30 days post-randomization. Our study protocol and patient information documents have been approved by the ethics committee of Guangdong Provincial People's Hospital [No. GDREC2019521H(R1)] and was registered in clinical trials (www.clinicaltrials.gov, NCT04491760). This study will be performed in compliance with the Code of Ethics of the 1964 Declaration of Helsinki and its later amendments. The study would comply with the Consolidated Standards of Reporting Trials Statements (CONSORT) (9), and provided CONSORT checklist when reported the study results.

### Study Oversight

A specific data safety committee has been guaranteed for the current research, consisting of two cardiologists and one statistician. The cardiologists will be responsible for the review of the in-hospital course and follow-up adverse events of the participants. Then, a safety analysis will be conducted by the statistician at the time the 20th participant is randomized, and the data will be divided into IMT-associated events or non-IMT-associated events.

### Study Population

The inclusion criteria are as follows: (1) age ≥ 18 years; patients with AMI undergoing primary PCI, admitted to the cardiac intensive care unit and at a high risk for pneumonia; and (2) be able to understand and agree with informed consent.

The exclusion criteria are: (1) a history of cerebrovascular accident; (2) have been treated with immunosuppressive medication for 30 days before the study; (3) have a neuromuscular disorder; (4) have cardiovascular instability (such as aortic dissection or unstable hemodynamics); (5) a history of coronary artery bypass grafting (CABG); (6) expected survival is < 6 months due to non-cardiogenic disease; (7) participated in other drugs or devices studies within 30 days; (8) confirmed ventricular aneurysm; and (9) other conditions not suitable for the study after the discussion among the researchers (e.g., poor compliance and unable to cooperate for the training).

After reviewing the results of previous studies and the clinical risk factors for pneumonia (1, 2, 7, 10), the following parameters will be used for pneumonia risk calculation: (1) age > 55 years; (2) history of diabetes mellitus, (3) present smoker; (4) chronic kidney disease [estimated glomerular filtration rate (eGFR) < 60 mL/min/1.73m^2^)]; and (5) forced expiratory volume in the first second of expiration (FEV_1_) <80% predicted and FEV_1_/forced vital capacity (FVC) <70% predicted. Having two or more of these parameters is regarded as being high risk for pneumonia.

### Randomization

**Figure 1:**
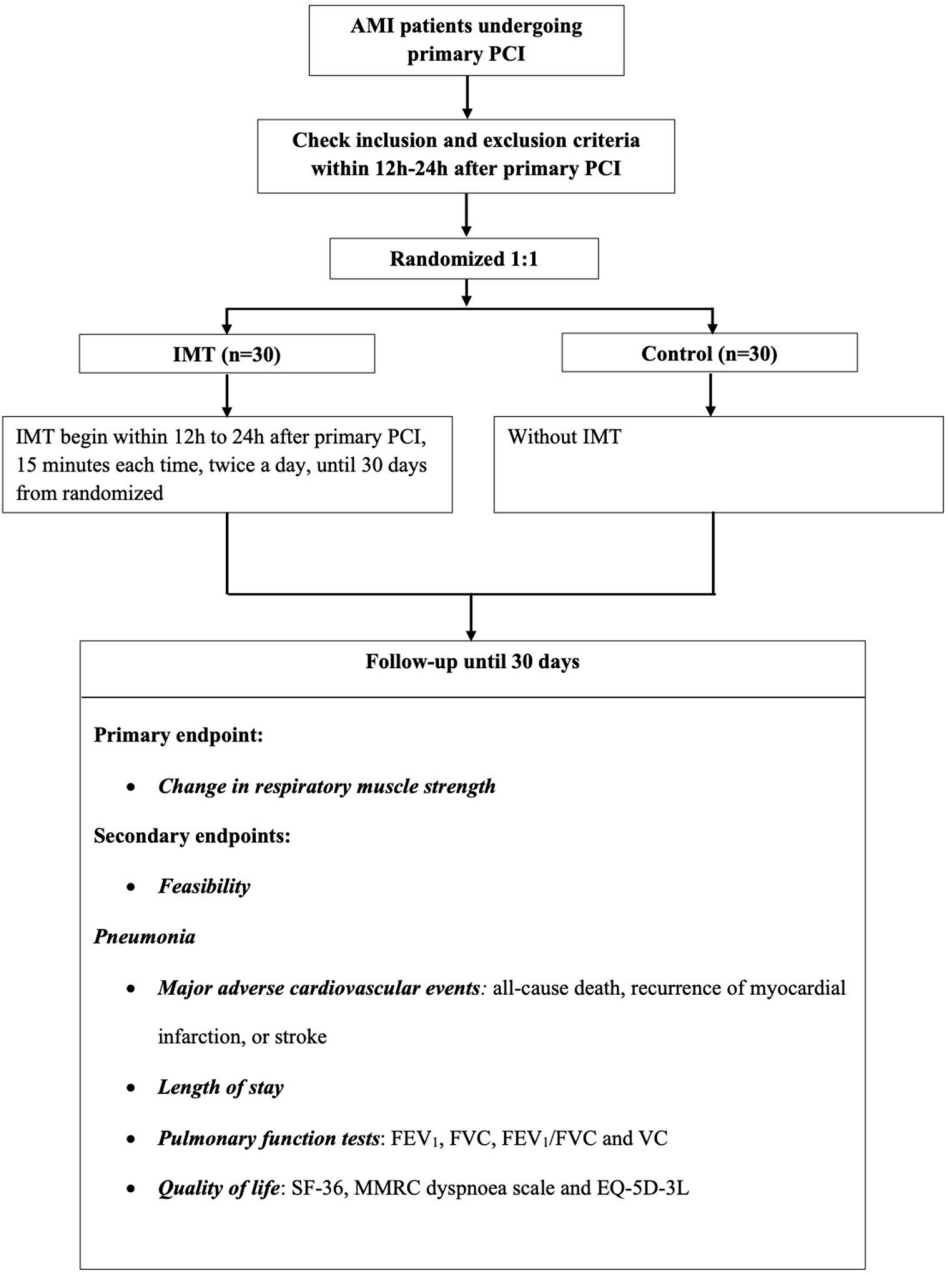
Study flow chart.

A specific computer-generated, randomized number will be placed in a sealed envelope. Researchers will be blinded to the information in envelopes and will select consecutive envelopes to distribute to consecutive participants, thus randomly assigning them to either the IMT group (*n* = 30) or the control group (*n* = 30). See study flow Figure 1.

### Invention: IMT

For the IMT group, IMT will be performed for 15 min *per session*, twice a day, with an initial load of 30% of MIP using a threshold inspiratory muscle trainer (c-type, Shengchang medical equipment factory, Yuyao City, China). During the hospital period, the resistance will increase incrementally, based on the rate of perceived exertion scored on the Borg scale (11). If the rate of perceived exertion is <5, the resistance of the inspiratory threshold trainer will then be increase incrementally by 5%. To facilitate training for participants, the load for home-based IMT training will be set at the highest training load achieved while the patient was in hospital. All of the participants will be required to complete maneuvers in the seated position during training, wearing nose-clips. IMT will be performed every day from 12 to 24 h after primary PCI and the final session will be performed 30 days post-randomization. Participants in the control group will receive standard care after primary PCI according to the current clinical practice guidelines (12), which included risk stratification, continuous monitoring and specific nursing. And we also encourage an early ambulation, based on the complications, symptoms, and capacity.

### Baseline Characteristics and Medicine

Electrocardiogram will be performed before primary PCI, and repeated testing will also be required. Transthoracic echocardiography will be performed within 24 h after primary PCI, mainly for early identification of cardiac rupture or ventricular aneurysm. X-ray and/or pulmonary computed tomography will be performed to diagnose pneumonia, and repetitive testing should be considered if necessary. A loading dose of antiplatelet agents will be given before coronary angiography and all of the catheter techniques will be performed according to guidelines (13). During the intervention procedure, intra-aortic balloon pump or other invasive equipment will be used if necessary. For the in-hospital stay, medications, such as antiplatelet agents, angiotensin-converting enzyme inhibitors, and β-blockers, will be prescribed by the cardiologists as needed.

### Follow-Up

Participants will be followed by two experienced researchers every week through phone interviews after discharge. Details of IMT will be documented, quality assessment will be performed, and any reported adverse events will be followed up, with assistance provided if necessary. All of the participants will be asked to return to the clinic for follow-up visits at 30 days post-randomization.

### Objectives

The aim is to assess the effectiveness of IMT by measures including the improvement of inspiratory muscle strength, pulmonary function, and quality of life, and by the avoidance of clinically adverse events. We will also assess the feasibility of IMT by examining the safety of training performance and through use of a satisfaction questionnaire.

### Study Endpoints

#### Primary Endpoint

##### Change in Inspiratory Muscle Strength

Static MIP will be selected for the estimation of inspiratory muscle strength. A standard measurement according to the *ERS Statement on Respiratory Muscle Testing at Rest and during Exercise* published by the European Respiratory Society (ERS) will be taken (14) (Powerbreathe® K5). The MIP test will be performed at baseline and at 30 days post-randomization.

#### Secondary Endpoints

##### Feasibility

Adverse events, heart rate, systolic blood pressure, diastolic blood pressure, and oxyhemoglobin saturation of patients during training while in hospital will be recorded. In addition, the workloads of MIP and the Borg scores will be recorded after every day of training in the hospital. The satisfaction of patients in the IMT group will be evaluated using a questionnaire at discharge and again at 30 days post-randomization (Supplementary Appendix 1).

##### Pneumonia

Pneumonia will be defined as the presence of a new or progressive radiographic infiltrate plus at least two of three clinical features (fever > 38°C, white blood cell count [WBC] > 10 × 10^9^/L or < 4 × 10^9^/L and purulent secretions), based on American Thoracic Society guidelines (15). Events of pneumonia will be followed and recorded by experienced doctors when participants are in hospital. After discharge, participants will be followed by experienced researchers and if a diagnosis of pneumonia is considered possible, guidance will be provided for the patients to complete necessary examinations, until 30 days post-randomization.

##### Major Adverse Cardiovascular Events (MACE)

MACE will be defined as all-cause death, recurrence of myocardial infarction, or stroke at 30 days post-randomization.

##### Length of Stay (LOS)

Length of stay will be assessed by calculating the number of days the patient is in the hospital.

##### Pulmonary Function Tests

Pulmonary function tests will be assessed by the following spirometry parameters: forced expiratory volume in the first second of expiration (FEV_1_), forced vital capacity (FVC), FEV_1_/FVC and vital capacity (VC), and reported as a percentage of the predicted value. A spirometer (microQuark, COSMED) will be used for these measurements, which will be performed at baseline and at 30 days post-randomization.

##### Quality of Life

Questionnaires including the 36-item short-form (SF-36) (16), Modified Medical Research Council (MMRC) dyspnoea scale (17) and EQ-5D-3L (18) will be used for the assessment of quality of life in our participants. Both interviews will be performed twice, first at baseline and again at 30 days post-randomization.

### Laboratory Testing

Routine clinical parameters, including laboratory tests such as electrolytes, troponin I/T, creatine kinase-MB (CK-MB), blood lipid, serum creatinine, and other clinical routine parameters examination will be performed within 12 h after admission. The eGFR will be assessed with the four-variable modification of diet in renal disease equation for Chinese patients (19). An additional 4 mL venous blood sample will be collected, and the isolated serum will be stored at −80°C for a future mechanism study.

### Assessment and Reporting of Adverse Events

Adverse events including all-cause death, recurrence of myocardial infarction, stroke, stent thrombosis, angina, dyspnea, acute heart failure, arrhythmia, and other unforeseeable incidents will be recorded in detail during both the in-hospital and follow-up periods. The reporting and documentation of adverse events will be classified as IMT-related or non-IMT-related. This safety analysis will be conducted at the end of our research among patients who had at least 1 day of outpatient safety data.

### Statistical Analysis

#### Sample Size Calculation

Our sample size calculation was based on a previous study (20). The mean of the MIP difference between the experimental group and the control group was estimated to be 15 cm H_2_O, the standard deviation was 12 cm H_2_O, the significance level was α = 0.05 and the test power was 1-β = 0.8. The resulting required sample size for each group was 11. Considering our projected 20% attrition rate, the sample size of each group should be increased to 14. To better study the secondary endpoints, a total of 60 cases will be included in the final analysis.

#### Data Analysis

Continuous data will be presented as mean ± standard deviation (SD) and we will use the Student's *t*-test or analysis of variance for comparisons between groups when the variables are normally distributed. Otherwise, we will present data as median and interquartile range and will use Wilcoxon rank-sum test for comparisons. Categorical data will be presented as percentages and χ^2^ test or Fisher's exact test will be selected for data comparison. For the study endpoints, pneumonia and MACE will be compared between the IMT group and the control group with the odds ratio (OR). Other endpoints will be compared between groups as appropriate. The SAS version 9.4 (SAS Institute, Cary, NC) will be used to perform our statistical analyses. *P* values will be 2-sided and *P* < 0.05 will be regarded as significant.

## Discussion

To the best of our knowledge, the proposed protocol is the first study to assess the safety and efficacy of IMT in patients with AMI undergoing primary PCI who are at a high risk of pneumonia during the acute period. Studies have rarely focused on the uncommonly high rate of pneumonia after AMI, so the potential benefits of IMT for these patients may have been neglected. We will focus on the improvement of inspiratory muscle strength after 30 days of training. In addition, we will record the clinical adverse events and the satisfaction of these patients and try to determine whether IMT has a potential benefit for decreasing incidence of pneumonia, MACE and length of stay (LOS), and also determine if IMT could benefit pulmonary function and quality of life.

For the consideration of pulmonary function test safety, the major evidence comes from one study, which reported the incidence of fatal cardiac events as 0.03%, and the incidence of other non-fatal events during testing as 1.49% and that symptom-limited protocols could increase the rate of major cardiac complications by 1.9 times when compared to submaximal tests (21). The incident risk was low, and the data were reported as a range; thus, the number of tests performed within 7 days after AMI was unknown. The previous guidelines suggested pulmonary function tests should not be performed within 30 days after AMI, whereas the new ERS guidelines regard AMI as a relative contraindication for pulmonary function testing (14, 22). Some scholars claimed that tests at 7 days after AMI deem to be safe (23). However, all of these suggestions were less evidence-based and further study should be performed. Although both focused inspiration and expiration could affect intrathoracic pressure, transmural pressure, and cardiac hemodynamics, whether they could result in clinical adverse events is unknown (24, 25). Two studies published in recent years have added to the safety data for use of pulmonary function tests in patients with AMI (8, 26) and the overall rates of mechanical complications are relatively lower in the primary PCI era (27), whereby fatal mechanical complications, such as ventricular rupture and left ventricular pseudoaneurysm, might require emergency surgery (28). In the present study, early transthoracic echocardiography will be performed to identify these patients. Adverse events, heart rate, systolic blood pressure, diastolic blood pressure, and oxyhemoglobin saturation of patients will be supervised. IMT has been demonstrated to be feasible and safe in patients who were undergoing CABG (7, 29–31), lung surgery (32), and those suffering from subacute stroke (33). By considering the above and rare IMT-related incidents that have been reported and excluding patients who might at high risk for adverse events due to pulmonary function testing, we believe that IMT for this cohort of patients with AMI might be relatively safe.

Previous studies have shown that IMT exceeding 2 weeks of therapy was effective for improving MIP (34), while the efficiency of implementing a shorter training time (< 10 days) still needs further investigation (29, 30, 35). As heart failure is common in patients with AMI, chronic heart failure would play an important role in the study cohort. The present recommendation for patients with heart failure participating in an IMT regimen is to begin with 30% MIP, then gradually increase to 60% MIP, 20–30 min *per session* with 3–5 exercise sessions per week (36). Some large-sample randomized trials have also confirmed the efficiency of these settings (31) and served as the basis of our IMT protocol design.

There are some limitations in our study. First, the present design is a single-center study, and the sample size is relatively small. Second, we selected guideline-based criteria for the diagnosis of pneumonia because no specific criteria have been developed for patients with AMI (15), and the criteria of the Centers for Disease Control and Prevention is not defined. AMI is associated with inflammatory reaction (37), and such a state might reduce the diagnostic power of most common clinical biomarkers. Although a new or progressive radiographic infiltrate and the presence of purulent secretions is the common diagnostic and prognostic indicator of pneumonia (31, 38–40), the significance of fever and white cell count may be limited by the inflammatory stage, which might lead to slightly overall excessive diagnoses (41–43). Third, the important aim of our study is to explore the role of IMT in preventing pneumonia. However, since no high-quality IMT study has been conducted in patients with AMI, we decided to calculate the sample size for our study using inspiratory muscle strength, and later extended this to better explore the effect of IMT on the secondary endpoints, especially pneumonia. Fourth, the model for calculating the risk of pneumonia was based on previous work in patients with ST-elevation myocardial infarction, without validation of its accuracy in our selected patient population (1, 2). Because there are not risk model for pneumonia in patients with AMI currently.

In conclusion, we have considered the significance of this proposed study protocol that was designed to determine the feasibility and potential clinical benefits of IMT in patients with AMI undergoing primary PCI who are at high risk of pneumonia, using a randomized, controlled trial.

## Ethics Statement

The studies involving human participants were reviewed and approved by the ethics committee of Guangdong Provincial People's Hospital. The patients/participants provided their written informed consent to participate in this study.

## Author Contributions

NT and YL contributed to the conception and design. YD, ZL, HZ, MZ, XC, and SZ searched the associated data. YD and YL drafted the manuscript. GZ, LX, CD, and JC provided the supervision support. LG, PH, and NT provided administrative support and performed data analysis. All authors contributed to the critical revisions and final approval of the manuscript.

## Conflict of Interest

The authors declare that the research was conducted in the absence of any commercial or financial relationships that could be construed as a potential conflict of interest.
