# Supplementary material for: The Safety and Efficacy of Inspiratory Muscle Training for Patients With Acute Myocardial Infarction Undergoing Percutaneous Coronary Intervention: Study Protocol for a Randomized Controlled Trial
Source: Front Cardiovasc Med. 2021 Jan 12;7:598054. doi: 10.3389/fcvm.2020.598054 (PMC7835280; doi:10.3389/fcvm.2020.598054)
Supplement: Supplementary file 1 [file Table_1.DOCX]

**Supplementary Appendix 1**

**eTable 1. Satisfaction assessment questionnaire**

**eTable 1. Satisfaction assessment questionnaire**

| **Please give the assessment for our inspiratory muscle training:** |
| --- |
| （1）How do you thinking about our inspiratory muscle training program?  Very good Good Common Not well |
| （2）How do you thinking about the intensity of our inspiratory muscle training?  Suitable High strength Low strength |
| （3）How do you thinking about the length of everyday inspiratory muscle training?  Suitable Too long Too short |
| （4）How do you thinking about your activity during inspiratory muscle training?  Quite active Common Really want to quit |
| （5）If you had another change, do you want to join in inspiratory muscle training again?    Willing Common Unwilling |
| （6）Did our inspiratory muscle training solve the clinical problem?  Yes No |
| （7）Is the period of inspiratory muscle training long enough?  Yes No |
| （8）Please give a score for our inspiratory muscle training (score: 1-10, score 1 means very poor, score 10 means very good ) |
| （9）Please give a satisfaction score for our inspiratory muscle training (score: 1-10, score 1 means very poor, score 10 means very good ) |
| （10）Please give a score for your activity about our inspiratory muscle training (score: 1-10, score 1 means very poor, score 10 means very good ) |
